# Acupuncture plus Chinese Herbal Medicine for Irritable Bowel Syndrome with Diarrhea: A Systematic Review and Meta-Analysis

**DOI:** 10.1155/2019/7680963

**Published:** 2019-04-14

**Authors:** Jing Yan, Zhi-wei Miao, Jun Lu, Fei Ge, Li-hua Yu, Wen-bin Shang, Li-na Liu, Zhi-guang Sun

**Affiliations:** ^1^Key Laboratory for Metabolic Diseases in Chinese Medicine, First Clinical Medical College, Nanjing University of Chinese Medicine, Nanjing 210023, China; ^2^Gastroenterology Department, Zhangjiagang TCM Hospital Affiliated to Nanjing University of Chinese Medicine, Suzhou 215600, China; ^3^Intensive Care Unit, Jiangsu Province Hospital of Traditional Chinese Medicine, the Affiliated Hospital of Nanjing University of Chinese Medicine, Nanjing 210029, China; ^4^Gastroenterology Department, Haian Hospital of Traditional Chinese Medicine, Nantong 226600, China; ^5^Gastroenterology Department, Wuxi Hospital of Integrated Traditional Chinese and Western Medicine, Wuxi 214000, China; ^6^Hepatology Department, the Affiliated Hospital of Nanjing University of Chinese Medicine, Nanjing 210029, China

## Abstract

**Purpose:**

To comprehensively evaluate the efficacy and safety of acupuncture combined with Chinese herbal medicine (CHM) in treating irritable bowel syndrome with diarrhea (IBS-D).

**Methods:**

Relevant randomized controlled trials (RCTs) were systemically retrieved from electronic databases from inception to March 2018, including the Cochrane Central Register of Controlled Trials (CENTRAL), PubMed, EMBASE, China National Knowledge Infrastructure (CNKI), Chinese Biological Medical Database (CBM, SinoMed), China Science and Technology Journal Database (VIP), and Wan Fang Data. Meanwhile, pooled estimates, including the 95% confidence interval (CI), were calculated for primary and secondary outcomes of IBS-D patients. Besides, quality of relevant articles was evaluated using the Cochrane Collaboration's risk of bias tool, and the Review Manager 5.3 and Stata12.0 softwares were employed for analyses.

**Results:**

A total of 21 RCTs related to IBS-D were included into this meta-analysis. Specifically, the pooled results indicated that (1) acupuncture combined with CHM might result in more favorable improvements compared with the control group (relative risk [RR] 1.29; 95% CI 1.24–1.35; P =0.03); (2) the combined method could markedly enhance the clinical efficacy in the meantime of remarkably reducing the scores of abdominal pain (standardized mean difference [SMD] –0.45; 95% CI –0.72, –0.17; P = 0.002), abdominal distention/discomfort (SMD –0.36; 95% CI –0.71, –0.01; P = 0.04), diarrhea (SMD –0.97; 95% CI –1.18, –0.75; P < 0.00001), diet condition (SMD –0.73; 95% CI –0.93, –0.52; P<0.00001), physical strength (SMD –1.25; 95% CI –2.32, –0.19; P = 0.02), and sleep quality (SMD –1.02; 95% CI –1.26, –0.77; P < 0.00001) compared with those in the matched groups treated with western medicine, or western medicine combined with CHM. Additionally, a metaregression analysis was constructed according to the name of prescription, acupuncture type, treatment course and publication year, and subgroup analyses stratified based on the names of prescriptions and acupoints location were also carried out, so as to explore the potential heterogeneities; and (3) IBS-D patients treated with the combined method only developed inconspicuous adverse events; more importantly, the combined treatment had displayed promising long-term efficacy.

**Conclusions:**

Findings in this study indicate that acupuncture combined with CHM is suggestive of an effective and safe treatment approach for IBS-D patients, which may serve as a promising method to treat IBS-D in practical application. However, more large-scale, multicenter, long-term, and high-quality RCTs are required in the future, given the small size, low quality, and high risk of the studies identified in this meta-analysis.

## 1. Introduction

Irritable bowel syndrome (IBS), a common functional gastrointestinal disorder, is characterized by recurrent abdominal pain or abdominal discomfort (the latter has been removed from the Rome IV criteria) and abnormal bowel habits [1]. According to one global meta-analysis, about 5%–22% of the general population has suffered from IBS [2], while such figure is 5%–10% in China [3]. Furthermore, the incidence of IBS shows a persistently increasing trend, which can be ascribed to the continuous development of modern society, the growing life/work pressure, and the changes in lifestyle and dietary structure. According to the Rome IV criteria [1], IBS patients can be subgrouped into IBS with diarrhea (IBS-D), IBS with constipation (IBS-C), mixed IBS (IBS-M), and IBS unclassifiable (IBS-U), among which IBS-D is the most frequently occurring subtype accounting for 40% [2]. IBS would cause no organic damage to the patient, but it would reduce the quality of life of the patient and consume a large amount of public healthcare resources [4, 5].

Currently, no generally accepted therapies are available to halt the progression of IBS, even though tremendous efforts have been made to uncover the mechanism of IBS. Besides, conventional pharmacotherapies (CP), such as antispasmodics, antidiarrheal agents, antidepressants, 5-hydroxytryptamine 3 (5-HT_3_) receptor antagonists, probiotics, and antibiotics, cannot achieve satisfactory clinical efficacy, and some of them are even associated with the risk of incidence of cardiovascular events and ischemic colitis [6].

Therefore, an increasing number of IBS patients have turned to alternative medicine, especially for traditional Chinese medicine (TCM), for symptom alleviation. Chinese herbal medicine (CHM) and acupuncture have long been practiced for a history of over two thousand years, which are recognized to be the most effective and popular therapies based on holistic concepts and syndrome differentiation of the TCM system. Some previous reviews regarding CHM [7, 8] or acupuncture [9] alone have suggested that both CHM and acupuncture have a beneficial effect on IBS-D symptoms. Nonetheless, no systematic review or meta-analysis is available at present to assess the effect of acupuncture combined with CHM on treating IBS-D. Therefore, the current study was designed aiming to improve the efficiency of statistical tests; compared with the evidence-based medical evidence from a single study, results in this study might prove to be more reliable for clinical practice and decision-making.

## 2. Materials and Methods

### 2.1. Retrieval Strategy and Study Selection

Relevant RCTs were systemically retrieved from the following databases: the Cochrane Central Register of Controlled Trials (CENTRAL), PubMed, EMBASE, China National Knowledge Infrastructure (CNKI), Chinese Biological Medical Database (CBM, SinoMed), China Science and Technology Journal Database (VIP), and Wan Fang Data, from their inception to March 2018 using the search terms of “irritable bowel syndrome,” “IBS,” “irritable colon,” “colon spasm,” “allergic colitis,” “colon allergy,” “irritable colon syndrome,” “acupuncture,” “acupuncture therapy,” “electroacupuncture,” “acupressure,” “ warm acupuncture,” “needling,” “needle warming moxibustion,” “auriculotherapy,” “traditional Chinese medicine,” “Chinese herbal medicine,” “herbal medicine,” “Chinese medicine,” and “complementary and alternative medicine.” S1 File has described an example from EMBASE in detail. Additionally, the reference lists from the included studies were also screened manually, so as not to leave out the potentially eligible trials.

Afterwards, the full-texts of all the eligible studies identified were reviewed, and study data were extracted by two researchers independently using a unified form. The original authors would be contacted for further information when the outcome data of relevant studies were unclear or missing. Any disagreement was resolved after discussion with the third author. Typically, the study inclusion criteria of this meta-analysis were as follows: (1) studies that employed clear diagnostic criteria of IBS-D, such as the Rome I–IV criteria; (2) randomized controlled trials; (3) studies in which participants in experimental group had received acupuncture (including electroacupuncture, needling, acupressure, auriculotherapy, or needle-warming moxibustion) combined with CHM (such as decoction, granules, capsule, pill, tablet, powder, or injection), while subjects in control group underwent unlimited treatment types (such as western medicine, western medicine combined with CHM, Chinese medicine alone, or acupuncture alone); and (4) studies published in Chinese or English language. Meanwhile, the study exclusion criteria were as follows: (1) studies focusing on IBS-C, IBS-M, or IBS-U; (2) duplicate publications; (3) studies enrolling participants with severe enteric disease or who developed heart failure, renal failure, or a malignancy during the study period; (4) commentary, editorial, experience introduction, conference article, review, graduation thesis, or case report; (5) nonhuman or animal studies; (6) studies in which there were intervention measures in treatment group other than acupuncture and CHM; (7) the articles had no clear outcome indexes, or incomplete data; and (8) studies whose conclusions had conflicted with the original data.

### 2.2. Data Extraction and Assessment of Risk of Bias

Data were extracted and assessed by two reviewers independently based on a unified form, and any disagreement was settled through discussion with a third reviewer. The extracted study data included first author, year of publication, criteria of western medicine and TCM, study population, age, number of participants in experimental and control groups, interventions, outcomes, follow-up, and duration. Moreover, the methodological quality was evaluated by two authors independently based on the Cochrane Collaboration's risk of bias tool [10].

### 2.3. Statistical Analysis

The Review Manager 5.3 and Stata12.0 softwares were applied in this meta-analysis. In addition, the pooled relative risk (RR) and 95% confidence interval (CI) were reported for the clinical efficacy rate, while standardized mean difference (SMD) and 95% CI were reported for the continuous variable data. The potential heterogeneity was assessed by chi-square test and the inconsistency index statistic (I^2^), respectively [11]. A random effects model would be applied to calculate the pooled RR in the presence of substantial heterogeneity (I^2^ > 50% or P < 0.05); otherwise, a fixed effects model would be employed [12]. Besides, sensitivity analysis was also conducted by sequentially eliminating one individual study each time to explore the underlying sources of heterogeneity. Additionally, the potential publication bias was assessed by applying the funnel plot and Egger's test [13], whereas the trim-and-fill analysis was conducted to identify the possible asymmetry and assess the robustness of conclusions.

## 3. Results

### 3.1. Literature Selection

**Figure 1:**
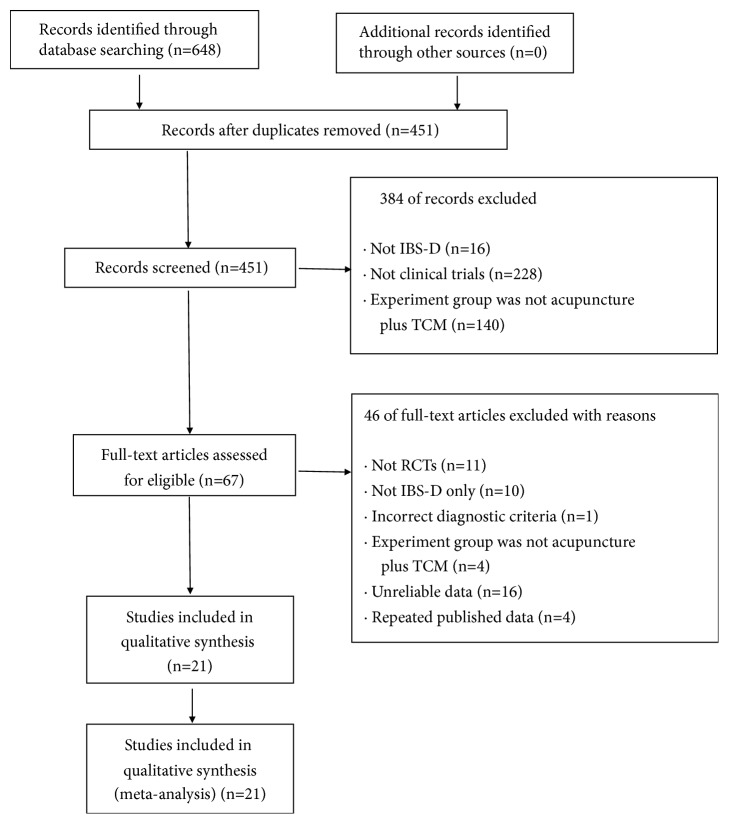
Flow chart of the process for literature retrieval.

A total of 648 studies were identified from the electronic bibliographic databases, among which 197 duplicate studies were removed. After reviewing the titles and abstracts, 384 studies were excluded since they were not qualified according to the predefined inclusion criteria. Then, the full-texts of the remaining articles were read, and 46 studies were excluded owing to non-RCTs (n = 11), including IBS-C or IBS-M subtypes (n = 10), incorrect criteria (n = 1), lack of relevant intervention treatment (n = 4), repeated published data (n = 4) and unreliable data (n = 16) including inconsistent data (the statistical results by the authors are inconsistent with the data in the literature, n = 10; average value of symptoms obtained in the results conflicted with the scoring criteria, n = 4; and the number of participants was inconsistent, n = 1), and incomplete data (the scoring criteria and symptom scores were not presented, n = 1). Eventually, 21 eligible studies were included into this systematic review and meta-analysis.  Figure 1 has described the detailed screening process for study identification and selection.

### 3.2. Study Characteristics

The study characteristics are presented in Tables 1, S1 and S2. As could be observed, a total of 21 RCTs involving 1,834 patients were enrolled (excluding 26 patients who had withdrawn or dropped out), including 929 receiving acupuncture combined CHM and 905 in control group [14–34]. All the eligible publications were written in Chinese and the studies were carried out in China. According to the western criteria, fourteen trials had utilized the Rome III criteria [20–25, 27–34], five had used the Rome II criteria [14–16, 18, 19], one had employed the Rome I criteria [17], and one had unknown diagnostic criteria [26]. On the other hand, 10 studies had described the TCM criteria in detail [16, 18, 21, 23–25, 28, 29, 32, 33], and the most common criterion was liver qi stagnation and spleen deficiency syndrome (LQSASDS), which was used in six trials [16, 18, 23–25, 28]. There was one multiple-center RCT [20] and 20 single-center RCTs [15–19, 22, 23, 25, 27, 28, 31, 32], with the sample size ranging from 30 to 102 and the mean patient age of 31–48 years (except that three trials reported only the age range [14, 15, 18] and three did not specify age [16, 19, 20]). Moreover, the treatment courses ranged from 2 weeks to 2 months, although one study did not report the treatment duration [16]. Besides, four trials had demonstrated follow-up that ranged from 4 weeks to 6 months [18, 23, 26, 31], and five had mentioned withdrawals and dropouts [21, 24, 31–33].

In terms of the dosage form in experimental group, 18 studies had used decoction [14–16, 18, 20–32, 34], while the remaining three had used the Chinese patent medicine [17, 19, 33]. With regard to the route of administration, 20 trials had adopted oral administration alone [14, 15, 17–34], whereas the rest one had combined oral with coloclysis application [16]. Among the prescriptions, Tongxie Yaofang (TXYF) [14, 15, 18, 21, 22, 24, 25, 28], a classical Chinese medicine prescription, was the most widely applied in relieving the IBS-D symptoms, followed by Xiaoyao San (XYS) [23, 26], Banxia Xiexin (BXXX) Decoction [29, 34] and Shenling Baizhu San (SLBZS) [31].

With regard to acupuncture, 13 trials had used conventional acupuncture alone [17, 19–22, 24, 25, 27–29, 32–34] (including two used mouth acupuncture [20, 27]), six had adopted conventional acupuncture combined with moxibustion [14–16, 18, 23, 26] (including four coupled with needle-warming moxibustion [14, 16, 23, 26]), and two had employed electroacupuncture therapy [30, 31]. Typically, the top six most commonly adopted acupoints were Zusanli (ST36), Tianshu (ST25), Taichong (LR3), Sanyinjiao (SP6), Shangjuxu (ST37), and Zhongwan (RN12). On the other hand, the therapeutic methods in control group included western medicine in 18 trials [14, 16–22, 24, 25, 27–34], among which the top two medicines were pinaverium bromide tablets (PBT) and montmorillonite powder (MP). In the meantime, the additional three trials had used western medicine combined with compound glutamine enteric-coated capsules (CGECC) [15, 23, 26].

In this meta-analysis, the primary outcome was the clinical efficacy rate, while the secondary outcomes were TCM symptom scores of abdominal pain, abdominal distension or discomfort, diarrhea, diet condition, physical strength, and sleep quality. Besides, adverse reactions and long-term efficacy were also recorded in this meta-analysis.

### 3.3. Methodological Quality

**Figure 2:**
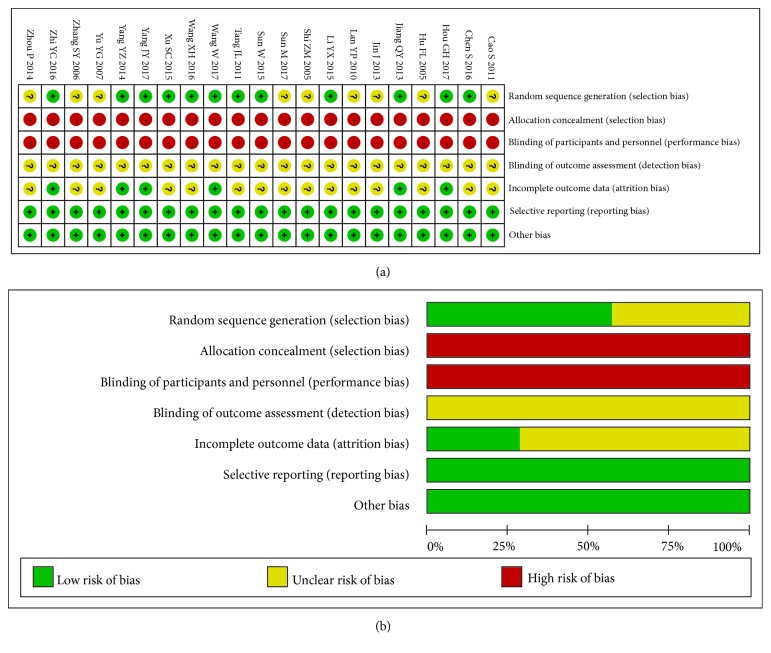
(a) Risk of bias summary. (b) Risk of bias graph.

Further information about some of the included trials was requested from the authors by e-mail; unfortunately, no response was received. Typically, ten RCTs had adopted a random number table to divide the participants into experimental group [20, 21, 24, 26, 27, 29–33] and control group, one had adopted a random form by shooting dice to allocate its participants [28], and one had applied the treatment order to divide its participants into two groups [25], while the remaining RCTs did not mention the specific randomization technique [14–19, 22, 23, 34]. Noticeably, no included trial had described the allocation concealment or blinding methods. In addition, five trials had mentioned withdrawals and dropouts because of intolerance to the taste of CHM, sudden illness, adverse events, and loss to follow-up [21, 28, 31–33]; nonetheless, none of them had conducted the intention-to-treat (ITT) analysis in the case of dropouts. Therefore, the validity of this review had displayed high risk, as presented in Figure 2.

### 3.4. Primary Outcome

#### 3.4.1. Clinical Efficacy Rate

A total of 20 RCTs had reported the clinical efficacy rate [14–19, 21–34], excluding the study by Tang et al. that did not mention it. [20]. According to the “Guiding Principle for Clinical Research of New Drugs of TCM” [35], the clinical efficacy was calculated as the comprehensive efficacy index (CEI): CEI(%) = (the number of patients with improved clinical symptoms after intervention divided by the total number of patients)×100%, which had been the most extensively applied in estimating the symptom curative effect of TCM in China.

**Figure 3:**
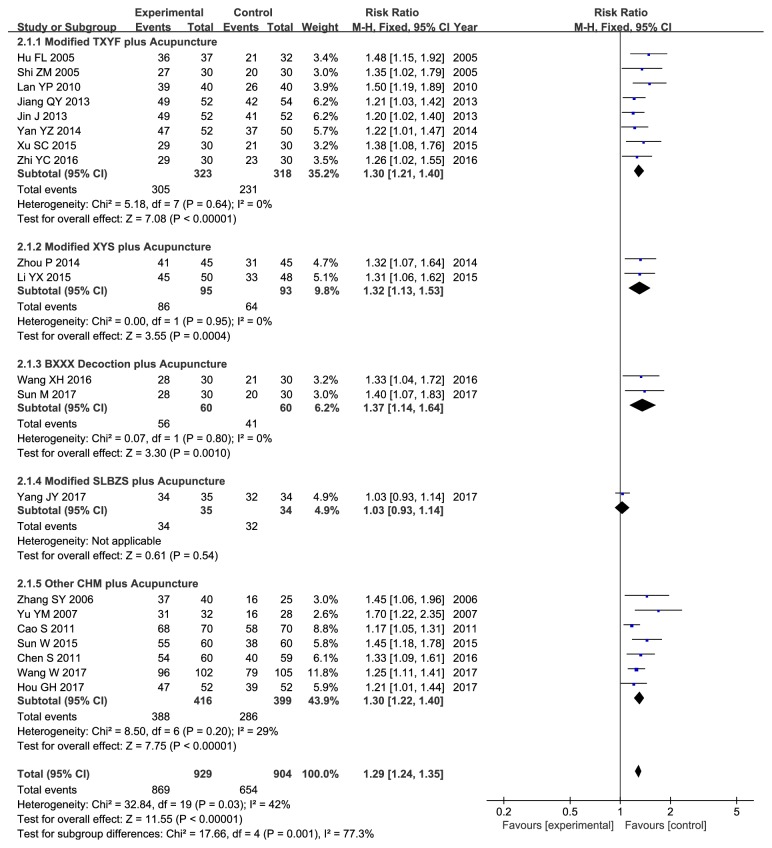
Forest plot of clinical therapeutic efficacy.

Our results indicated that acupuncture combined with CHM could enhance the clinical effectiveness compared with the control group treated with western medicine alone or western medicine combined with CHM (RR 1.29, 95% CI 1.24–1.35, P < 0.00001); however, mild heterogeneity was discovered (*χ* ^2^ = 32.84, P = 0.03, I^2^ = 42%) (Figure 3). Therefore, metaregression and subgroup analyses were used to explore heterogeneity.

The metaregression analysis was performed with the name of prescriptions, acupuncture type, treatment course, and publication year as independent variables; the results indicated that the heterogeneity could not be well explained (Table 2).

Subgroup analysis stratified based on the name of prescriptions was further conducted to identify the underlying source of heterogeneity, including Tongxie Yaofang (TXYF), Xiaoyao San (XYS), Banxia Xiexin (BXXX) decoction, and Shenling Baizhu San (SLBZS) that were derived from ancient time, as well as other CHM containing the self-ordained TCM decoctions and Chinese patent medicines formed in modern time. Compared with the control groups, the experimental groups had displayed favorable effects on boosting the clinical efficacy rate for the modified TXYF combined with acupuncture (RR 1.30, 95% CI 1.21–1.40, P < 0.00001) in eight trials with no heterogeneity (*χ* ^2^ = 5.18, I^2^ = 0%, P = 0.64) [14, 15, 18, 21, 22, 24, 25, 28], the modified XYS combined with acupuncture (RR 1.32, 95% CI 1.13–1.53, P = 0.0004) in two trials with no heterogeneity (*χ* ^2^ = 0.00, I^2^ = 0%, P = 0.95) [23, 26], the BXXX decoction combined with acupuncture (RR 1.37, 95% CI 1.14–1.64, P = 0.0010) in two trials with no heterogeneity (*χ* ^2^ = 0.07, I^2^ = 0%, P = 0.80) [29, 34], and other CHM combined with acupuncture (RR 1.30, 95% CI 1.22–1.40, P < 0.00001) in seven trials with low heterogeneity (*χ* ^2^ = 8.50,, I^2^ = 29%, P = 0.20) [16, 17, 19, 27, 30, 32, 33] (Figure 3). Sensitivity analysis suggested that the primary outcome would not be affected by sequentially omitting one individual study each time.

### 3.5. Secondary Outcomes

#### 3.5.1. Abdominal Pain

**Figure 4:**
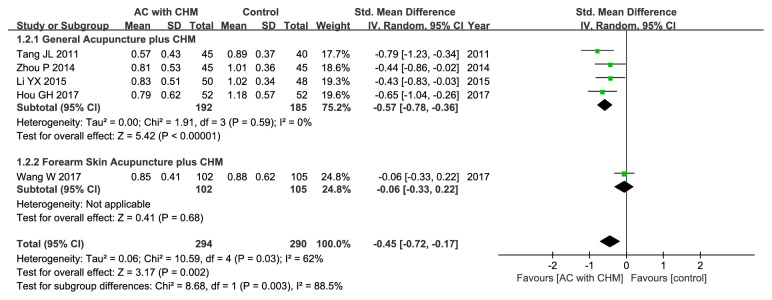
Forest plot of improvement in abdominal pain.

Five out of the included trials had evaluated the improvement of abdominal pain based on the symptom scores [20, 23, 26, 32, 33]. Our findings indicated that acupuncture combined with CHM could lead to greater alleviation of abdominal pain compared with the controls (SMD –0.45; 95% CI –0.72, –0.17; P = 0.002), and moderate statistical heterogeneity could be identified (*χ* ^2^ = 10.59, I^2^ = 62%, P = 0.03) (Figure 4). As a result, subgroup analysis stratified based on the location of acupoints was also carried out, which revealed that general acupuncture combined with CHM could remarkably alleviate abdominal pain relative to control group (SMD –0.57; 95% CI –0.78, –0.36; P<0.00001), with no heterogeneity (*χ* ^2^ = 1.91, I^2^ = 0%, P = 0.59) (Figure 4).

#### 3.5.2. Abdominal Distention or Discomfort Score

**Figure 5:**
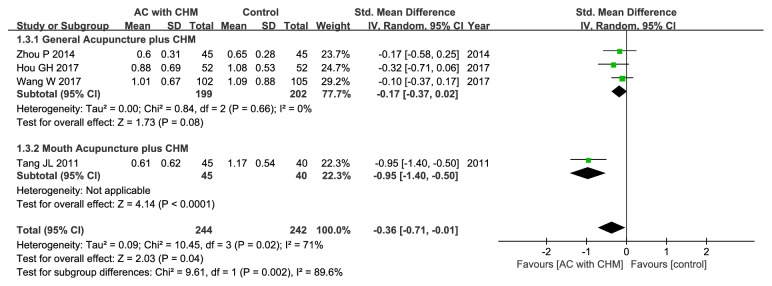
Forest plot of improvement in abdominal distention or discomfort score.

Three studies had assessed the abdominal distention score [20, 23, 32], while one had mentioned the abdominal distention or discomfort score [33], all of which were pooled to evaluate the effect on improving abdominal distention/discomfort. Compared with control group, the treatment group could favorably affect abdominal distention/discomfort (SMD –0.36; 95% CI –0.71, –0.01; P = 0.04), but significant heterogeneity could be detected (*χ* ^2^ = 10.45, I^2^ = 71%, P = 0.02) (Figure 5). On this account, subgroup analysis stratified based on the location of acupoints was also carried out, which suggested that general acupuncture combined with CHM could greatly relieve abdominal distention/discomfort relative to control group (SMD –0.17; 95% CI –0.37, –0.02; P = 0.08) with no heterogeneity (*χ* ^2^ = 0.84, I^2^ = 0%, P = 0.66) (Figure 5).

#### 3.5.3. Diarrhea

**Figure 6:**
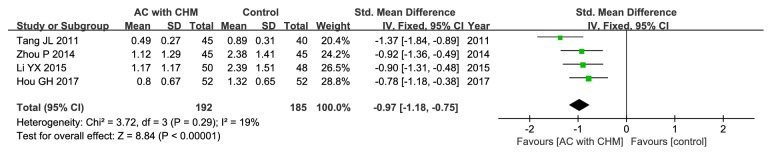
Forest plot of improvement in diarrhea score.

Four out of the included studies had evaluated the diarrhea score [20, 23, 26, 33], and the pooled results showed that the experimental group could greatly improve diarrhea compared with control group (SMD –0.97; 95% CI –1.18, –0.75; P < 0.00001), and low heterogeneity could be identified (*χ* ^2^ = 3.72, I^2^ = 19%, P = 0.29) (Figure 6).

#### 3.5.4. Diet Conditions.

**Figure 7:**
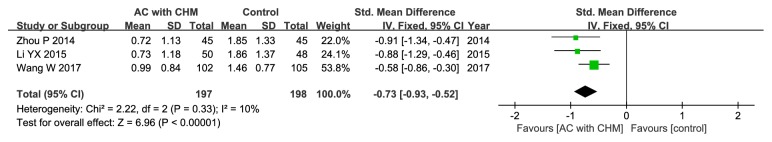
Forest plot of improvement in the diet condition score.

Three of the 21 studies had mentioned the outcome of diet improvement [23, 26, 32], all of which were thus incorporated into this meta-analysis. Our findings displayed that acupuncture combined with CHM could greatly improve the diet conditions compared with control groups (SMD –0.73; 95% CI –0.93, –0.52; P < 0.00001), and low statistical heterogeneity could be detected (*χ* ^2^ = 2.22, I^2^ = 10%, P = 0.33) (Figure 7).

#### 3.5.5. Physical Strength.

**Figure 8:**
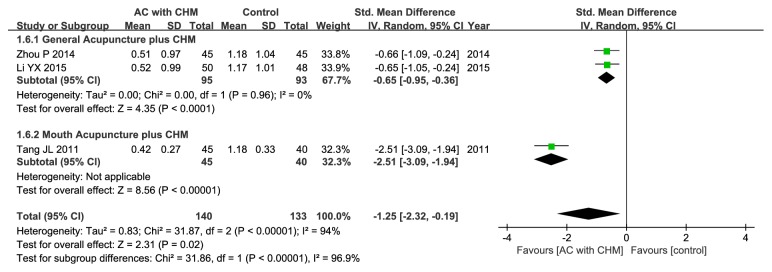
Forest plot of improvement in physical strength score.

Three out of the included trials had discussed the outcome of physical strength [20, 23, 26], and better effect could be attained in treatment group relative to the controls (SMD –1.25; 95% CI –2.32, –0.19; P = 0.02). Unfortunately, there was significant heterogeneity (*χ* ^2^ = 31.87, I^2^ = 94%, P < 0.00001) (Figure 8); as a result, subgroup analysis was performed, which suggested that general acupuncture combined with CHM could greatly alleviate physical strength compared with control group (SMD –0.65; 95% CI –0.95, –0.36; P<0.0001), with no heterogeneity being detected (*χ* ^2^ =0.00, I^2^ = 0%, P=0.96) (Figure 8).

#### 3.5.6. Sleep Quality

**Figure 9:**
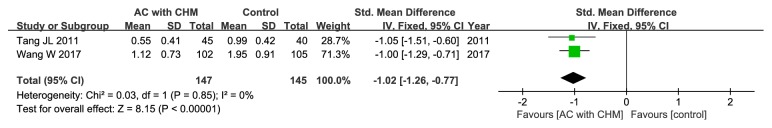
Forest plot of improvement in sleeping quality score.

Two of the enrolled articles had assessed the sleep quality [20, 32], and the pooled data demonstrated that the experimental group could achieve greater improvement in sleep quality (SMD –1.02; 95% CI –1.26, –0.77; P < 0.00001), with no heterogeneity in the data (*χ* ^2^ = 0.03, I^2^ = 0%, P = 0.85) (Figure 9).

#### 3.5.7. Safety Profile and Adverse Events

A total of eight enrolled studies had evaluated the safety profile [21, 22, 24, 26, 28, 31–33]. Typically, no severe adverse events were observed in treatment group, even though two mild cases were reported in one study [22] (including one with nausea and vomiting and the other one with headache) and two cases of stomach upset were mentioned in another study [21]. Fortunately, the symptoms in these cases had disappeared after the diet was changed and all patients had accomplished the course of treatment. However, some patients could not tolerate the taste of CHM and had withdrawn from the trial [32]. Additionally, four trials had reported drug withdrawal events among the control group, including one for abdominal pain in the study by Zhi et al. [28], one due to skin rash by the studies of Yang et al. [31] and Jiang et al. [21], and three as a result of unmentioned manifestation in the study by Wan et al. [32]. Meanwhile, four cases with nausea, dizziness, fatigue, and sleepiness were reported by Yang et al. [31], two side effects (including one of erythra and the other one of pruritus) were mentioned by Yan et al. [24], and two patients suffering from pruritus were presented by Hou et al. [33]; however, all of these patients could tolerate the effects and had completed the treatment course. Moreover, in the study reported by Li et al. [26], no untoward effect was observed in both experimental and control groups.

### 3.6. Long-Term Observations

Among all enrolled studies, two trials had evaluated the long-term curative effect [18, 31]. For instance, Lan et al. reported that the recurrence rate in treatment group was 10% at follow-up examination 6 months upon the completion of the treatment course, which was dramatically lower than that in control group (71%) [18]. Besides, Yang et al. had assessed the irritable bowel syndrome symptom severity score (IBS-SSS), irritable bowel syndrome quality of life (IBS-QOL), and symptom check list-90 (SCL-90) during follow-up 4 weeks after the completion of treatment course, but they had not described the clinical efficacy rate [31]. Their results indicated that acupuncture combined with CHM could evidently improve the clinical symptoms and psychological status of IBS-D patients. Additionally, one study had described the long-term side effects, which revealed no difference between the two groups [26], and another one study had mentioned the long-term observation, but no details were provided [23].

### 3.7. Publication Bias

**Figure 10:**
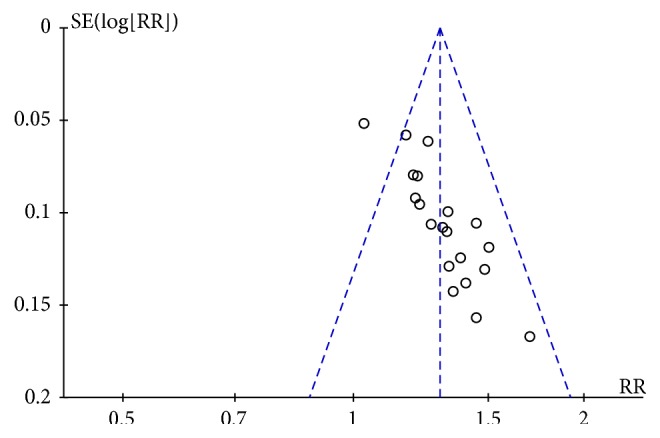
Funnel plot of publication bias.

Figure 10 and S2 File revealed that possible publication bias could be detected through observing the asymmetrical plot and Egger's test (P = 0.000). Furthermore, trimming was required for nine additional studies based on trim-and-fill analysis, with the pooled estimate of 1.205 (95% CI 1.136–1.273) after filling, indicating that the conclusion would not be affected the publication bias (S2 File).

## 4. Discussion

### 4.1. Summary of Evidence

Altogether 21 RCTs related to IBS-D were included into the current meta-analysis, and the pooled results demonstrated that (1) acupuncture combined with CHM had exhibited favorable improvement compared with the controls; (2) compared with the matched groups treated with western medicine or western medicine combined with CHM, the combined method could markedly enhance the clinical therapeutic efficacy in the meantime of reducing the scores for abdominal pain, abdominal distention/discomfort, diarrhea, diet condition, physical strength, and sleep quality; (3) IBS-D patients treated with the combined method would not suffer from obvious adverse events and promising long-term efficacy could be attained by the combined method; and (4) the low methodological quality of the enrolled articles would give rise to evidence of publication bias, which should be further improved in future studies.

### 4.2. Significance of the Study

IBS is a common disease, but no universally accepted therapy is available at present to halt its progression. Hence, increasing patients and practitioners have turned to acupuncture combined with CHM for treatment. Typically, acupuncture contributes to achieving obvious and fast effect without causing adverse reaction; however, it is limited by its action area and span. As is well known, CHM has comprehensive and long-lasting effects, but some medicine-related side effects have been reported [36]. It is suggested in some meta-analyses that acupuncture or CHM alone can attain a more satisfactory clinical efficacy rate, greater improvement of specific symptoms (such as abdominal pain, diarrhea, and abdominal distension), while fewer obvious side effects than the control treatments [7–9]. Moreover, acupuncture combined with THM has displayed a synergistic effect on strengthening the curative effect, shortening the course of treatment, prolonging the duration of efficacy, and reducing side effects. Results of our meta-analysis indicated that the combined method could improve the above-mentioned factors and greatly alleviate the general symptoms, especially those related to physical strength, sleep, and diet, which could hardly be achieved through western medicine. However, few RCTs have studied the similarities and differences among acupuncture combined CHM, acupuncture alone, and CHM alone [20]; as a result, more studies are required to more comprehensively investigate the superiorities of the combined method. On the other hand, there were also some conflicts in the data; for example, 19 of the enrolled trials reported that the combined method could achieve greater clinical therapeutic efficacy than the control treatments [14–19, 21–30, 32–34], but Yang et al. suggested that there was no obvious difference between these different treatments [31]. Nonetheless, the pooled data revealed that acupuncture combined with CHM might potentially be the more appropriate means for treating IBS-D. Besides, there were also some paradoxical effects on individual symptoms; for instance, no significant difference was found in abdominal pain [32] or abdominal distention in some studies [23, 32], but the pooled data had indicated a positive result. Three trials had reported abdominal distention [20, 23, 32] and one had mentioned abdominal distention or discomfort [33], but abdominal discomfort was multifaceted in IBS, which might involve a range of symptoms like bloating, fullness, and flatulence [37]. As a result, these four trials were incorporated into analysis to enhance the reliability of results. Hence, our findings indicated that acupuncture combined with CHM had a superior curative effect on diarrhea and quality of life, as well as on abdominal pain and abdominal distention/discomfort, revealing that the combined treatment was a more efficient way to treat IBS-D patients.

In addition, the therapeutic schedules were different among the 21 included trials, and the most appropriate combination of acupuncture and CHM could be formulated through systemic analysis to achieve better therapeutic results. In acupuncture, traditional Chinese physicians prefer to apply needles without using electroacupuncture apparatus. Typically, the top six most commonly used acupoints were Zusanli (ST36), Tianshu (ST25), Taichong (LR3), Sanyinjiao (SP6), Shangjuxu (ST37), and Zhongwan (RN12), which had played an important and synthesized role in invigorating the spleen, resisting diarrhea, relieving the liver, and alleviating pain. Additionally, the most commonly adopted meridians were the Stomach Meridian of Foot-Yangming, the Liver Meridian of Foot-Jueyin, the Spleen Meridian of Foot-Taiyin, the Ren Meridin, and the Bladder Meridian of Foot-Taiyang. Typically, acupoints were mainly selected based on Zang Fu organs and meridians, which were often joined in the following ways, he-sea point matching front Mu point, back-shu point matching front Mu point, and back-shu point matching he-sea point; for instance, Zusanli (ST36) and Zhongwan (RN12), Pishu (BL20) and Tian shu (ST25), or Pishu (BL20) and Zusanli (ST36).

In the field of CHM, 18 among the enrolled RCTs had applied a Chinese herbal decoction [14–16, 18, 20–32, 34], which was regarded as the preferred choice when focusing on holistic therapy as well as syndrome differentiation and treatment. Besides, the classical Chinese prescriptions, including TXYF, XYS, and BXXX decoctions, were commonly prescribed to relieve the IBS-D symptoms. In particular, TXYF is a representative formula for reconciling liver and spleen, which has been modified by a majority of herbalists to treat IBS-D. In this review, nearly half of the enrolled trials had utilized this prescription [14, 15, 18, 21, 22, 24, 25, 28, 31]. Furthermore, it was worth noting that enteroclysis of CHM might also be a potential therapy for IBS-D patients, although such method was only reported in one study [16].

More importantly, this was the first systematic review and meta-analysis to assess the efficacy of acupuncture combined with CHM in treating IBS-D, which was also the first to assess the scores for diet condition, physical strength, and sleep quality compared with other meta-analyses evaluating the effect of CHM or acupuncture on IBS-D [7–9]. Specifically, the above-mentioned factors could provide guidance for clinicians to treat IBS-D.

### 4.3. Potential Mechanism of Acupuncture and CHM

Acupuncture combined with CHM can achieve a satisfactory synergistic effect, which may be ascribed to the following aspects [38]. (1) It can effectively regulate the internal organs through the nervous, endocrine, and immune systems; particularly, it can increase the excitability of target organ and expand the microvascular circulation, thus augmenting the amount of perfused target organ per unit time. (2) It can also affect the receptors on corresponding organ cell surface to upregulate the expression of receptors that are compatible with CHM, thereby enhancing the affinity between the affected organ cells and CHM. Notably, such process may be the main factor affecting the synergies between acupuncture and CHM. The pathogenesis of IBS-D remains unclear, and no existing study has revealed the mechanism of acupuncture combined with CHM in IBS-D. Nevertheless, many experiments have been carried out attempting to uncover the underlying mechanisms from different perspectives, such as [39–43] (a) inhibiting the hyperactive intestinal motility, (b) reducing the visceral hypersensitivity, (c) relieving the low level intestinal mucosal inflammation, (d) regulating the brain-intestine axis, (e) mitigating enteric dysbacteriosis, and (f) resisting depression.

Yuan et al. had demonstrated that TXYF could inhibit the colonic hypermotility through preventing the influx of extracellular Ca^2+^ into the isolated rat colonic smooth muscle cells [44]. In addition, it is also suggested in experiments that TXYF can alleviate the behavioral hyperalgesia, which also shows antidiarrheal activity related to inhibiting the activated intestinal mucosal mast cells, downregulating the expression of protease activated receptor-2 (PAR-2) and oncogene fos (c-Fos), and lowering the levels of TNF-*α* and IL-6 in rats with postinfective IBS [45, 46]. In addition, TXYF is also found to decrease the levels of gastroenteric hormones, such as 5-HT, substance P (SP), vasoactive intestinal peptide (VIP), somatostatin (SS), and cholecystokinin (CCK) in rats and humans with IBS [47, 48]. As for XYS, studies have revealed that XYS may raise the pain threshold and suppress the intestinal sensitivity through downregulating the expression of 5-HT_3_ receptors, upregulating that of 5-HT_4_ receptors, and lowering the serum cortisol level [49, 50]. Additionally, XYS can also alleviate gut dysbiosis to normal level in functional dyspepsia with liver depression-spleen deficiency syndrome, which is achieved through reducing the relative abundance of Firmicutes, Proteobacteria, and Cyanobacteria, whereas increasing that of Bacteroidetes [51]. Nonetheless, further studies should be carried out to more precisely discover the mechanisms of action of CHM in IBS.

Besides, other studies have also uncovered the mechanism of action of acupuncture in treating IBS-D, and experimental data show that electroacupuncture at Zusanli can also inhibit the vimentin protein expression level to adjust the gastrointestinal motility [52]. Additionally, the antidiarrheal effect has been connected to the regulation of the excitation of sympathetic nerves, which in turn will reduce the number of enterochromaffin cells (ECs), colonic tryptophan hydroxylase (TPH) expression, and 5-HT content in feces and colonic tissues in IBS-D rats [53]. Furthermore, Liu et al. had stimulated the Zusanli and Shangjuxu acupoints to lower the level of spinal N-methyl-D-aspartic acid (NMDA) receptor NR2B subunit in IBS rats, so as to counter the visceral sensitivity [54].

### 4.4. Limitations

The current meta-analysis was inevitably associated with some limitations, as displayed below.

(1) Geographical distribution: the combined method required that clinicians should master acupuncture and prescribe the Chinese medicine prescriptions expertly, which would inevitably lead to a geographically limited distribution of studies; therefore, the studies included in this meta-analysis were all conducted in China and published in the Chinese language.

(2) Poor methodological quality: only ten of the enrolled studies had employed a random number table for participant grouping [20, 21, 24, 26, 27, 29–33] and five studies had reported withdrawals and dropouts [21, 28, 31–33]; however, ITT analysis was not performed. Additionally, no study had mentioned random sequence generation, allocation concealment, blinding of participants and personnel, or blinding of outcome assessments. Moreover, there was only one multicenter trial [20], and a majority of the included studies had a small sample size.

(3) Evident heterogeneity: although the intervention and control groups were strictly incorporated into this meta-analysis, there was still moderate or high heterogeneity. Consequently, metaregression and subgroup analyses were performed to search for the potential source of heterogeneity. For metaregression analysis, it could not well explain heterogeneity. Instead, for subgroup analyses, with regard to clinical efficacy rate, the study by Yang et al. might account for one main source of significant heterogeneity [31] (Figure 3). All the included studies were carefully reviewed, and difference was found in the Chinese formula compositions between patients enrolled in the study by Yang et al. and those recruited in the other selected studies. In this study [31], the authors suggested that deficiency of spleen and kidney Yang also exerted a crucial part in the development of IBS-D in addition to liver depression and spleen deficiency. Therefore, Ganjiang (*Zingiberis Rhizoma*) and Rougui (*Cinnamomi Cortex*) were added to strengthen the Yang warming effect, which might be one of the leading causes of high heterogeneity. In terms of the secondary outcomes, the location of special acupoints could be an important source of high heterogeneity. Concretely speaking, Wang et al. had applied forearm skin acupuncture combined with CHM to treat abdominal pain [32] (Figure 4), and the acupoints selected in the forearm skin (inner skin of the upper arm from the wrist to the elbow) had included Wei acupoint (no international code), Xin acupoint (no international code), Gan acupoint (no international code), and Pi acupoint (no international code), which were different from the traditional acupoints and might thereby lead to different therapeutic effects. As far as abdominal distention/discomfort and physical strength were concerned (Figures 5 and 8), the high heterogeneity might be related to the study by Tang et al. which had adopted mouth acupuncture [20]. Typically, such acupuncture therapy was distinct from those employed in other studies, which might be ascribed to the fact that acupoints Shenjingqu (no international code) and Toubuqu (no international code) were in oral cavity and might result in different curative effects. Furthermore, there might be other possible reasons; for instance, (a) various forms of the combined methods, such as different compositions, dosage forms, and needling points; (b) different diagnostic criteria, including western criteria varied from Rome I to IV and TCM criteria involving different kinds of syndromes; and (c) the sketchy symptom grading criteria, for instance, only one trial [32] had described the symptom score criteria in detail, while the remaining had not. Therefore, it could hardly judge whether the researchers had used the same diagnostic criteria, which might possibly generate the potential assessment bias, although all of them had consulted the same publications as well as the Guiding Principle of Clinical Research on New Drugs of Traditional Chinese Medicine (Trial) [35].

(4) Potential evidence of publication bias: a majority of the enrolled studies in this meta-analysis had a small sample size and had reported a negative result, which might account for the major source of publication bias.

(5) Short-term interventions and follow-up: the treatment duration in most of the included studies was 4 weeks, and only four studies had mentioned follow-up [18, 23, 26, 31] for a period ranging from 4 weeks to 6 months. However, IBS is a chronic recurrent disease, and adequate treatment duration and follow-up periods should be included in the observations of studies.

## 5. Conclusions

To the best of our knowledge, this is the first systematic review and meta-analysis of acupuncture combined with CHM in treating IBS-D. The results indicate that the combined method is suggestive of an effective and safe therapy, which may serve as a promising method to treat IBS-D in practical application. However, the included studies of this meta-analysis are associated with poor methodological quality and heterogeneity in diagnostic and evaluation criteria, as well as in interventions of acupuncture and CHM; consequently, further rigorously designed, multicenter, and large-scale clinical RCTs are required to overcome the limitations of the current study and to enhance the strength of evidence.

## Figures and Tables

**Table 1:** Characteristics of included literature.

Source	Criteria		Study population	Age (years)		N (EG/CG)	Intervention		Outcomes	Follow-up	Side effects
	Western	TCM		EG	CG		EG	CG			
Shi ZM 2005	RomeII	N.M	Single center	20-55	22-58	30/30	Modified Sini Decoction and TXYF + Acupuncture	OT+DT+BT+MP	A	N.M	N.M
Hu FL 2005	RomeII	N.M	Single center	22-68, mean:37	20-60, mean:35	37/32	Modified TXYF+ Acupuncture	CGEC+MP+OT	A	N.M	N.M
Zhang SY 2006	RomeII	LQSASDS	Single center	N.M	N.M	40/25	Oral: Modified Chaishao Yigong and Xiangsha Pingwei Decoction, Enema: Self-ordained TCM Decoction + Acupuncture	LHS+MP	A	N.M	N.M
Yu YG 2007	RomeI	N.M	Single center	44	43	32/28	Chang Ling capsule + acupuncture	TDC	A	N.M	N.M
Lan YP 2010	RomeII	LQSASDS	Single center	18-62	19-61	40/40	Modified TXYF+ Acupuncture	MP+OT	A+O	6 months	N.M
Cao S 2011	RomeII	N.M	Single center	N.M	N.M	70/70	HXZQ Pill, or HXZQ Capsule, or HXZQ oral liquid + Acupuncture	RP+MBND	A	N.M	N.M
Tang JL 2011	Rome III	N.M	Two centers	N.M	N.M	N.M	Jianpi Huazhuo Tongluo Decoction + Acupuncture	LBP	C+D+F+H+K+M+N+S	N.M	N.M
Jiang QY 2013	Rome III	LSAASS	Single center	39.56±11.32	37.48±13.79	52/54	Chaihu Shugan Decoction + Acupuncture	OBT	A	N.M	T: 2 cases, C: 1 case
Jin J 2013	Rome III	N.M	Single center	42.52±3.73	42.94±3.81	52/52	Modified TXYF+ Acupuncture	RP, MBND	A+ T	N.M	T: 2 cases, C: 3 cases
Zhou P 2014	Rome III	LQSASDS	Single center	16~64, mean:39.3	17~65, mean:38.9	45/45	Modified XYS+ Acupuncture	MP+CGEC+CAECT	A+D+F+L+N+U	12 weeks	N.M
Yan YZ 2014	Rome III	LQSASDS	Single center	38.25	36.75	52/50	Modified TXYF+ Acupuncture	PBT+CBLEABCT	A	N.M	C: 3 cases
Xu SC 2015	Rome III	LQSASDS	Single center	42.3	40.9	30/30	Modified TXYF+ Acupuncture	PBT	A	N.M	N.M
Li YX 2015	N.M	N.M	Single center	41.1±7.87	40.2±8.56	50/48	Modified XYS + Acupuncture	MP+CGEC	A+C+F+L+N	12 weeks	no
Sun W 2015	Rome III	MBND	Single center	31.5±7.4	32.8±8.1	60/60	Jianpi Huazhuo Tongluo Decoction + Acupuncture	PBT	A	N.M	N.M
Zhi YC 2016	Rome III	LQSASDS	Single center	42.4±7.4	41.8±7.3	30/30	Dunhuang Baoyuan Decoction + Acupuncture	PBT	A	N.M	C: 1case
Wang XH 2016	Rome III	CCSS	Single center	38.93±5.98	39.40±7.94	30/30	BXXX Decoction + Acupuncture	PBT	A+B	N.M	N.M
Chen S 2016	Rome III	N.M	Single center	38.21±10.65	40.07±9.38	60/59	Shugan Jianpi Zhixie Decoction + Acupuncture	PBT	A+B	N.M	N.M
Yang JY 2017	Rome III	N.M	Single center	48.20±4.59	47.49±5.23	35/35	Modified SLBZS+ Acupuncture	TMT	A+P+Q+R	4 weeks	C: 5cases
Hou GH 2017	Rome III	LSAASS or WOSASS	Single center	36.75	37.55	52/52	CGECC+ Acupuncture	SB+PBT	A+C+D/E+F	N.M	C: 2 cases
Wang W 2017	Rome III	DBLASS	Single center	35.2±4.3	36.2±5.0	102/105	Self-ordained TCM Decoction + Acupuncture	PBT	A+B+C+D+G+L+M	N.M	C: 3 cases
Sun M 2017	Rome III	MBND	Single center	38.79±5.89	38.65±5.76	30/30	BXXX Decoction + Acupuncture	PBT	A+B	N.M	N.M

Annotation:

EG: experiment group; CG: control group; N.M: not mentioned; MBND: mentioned but not described;

CCS: cold-heat complicated syndrome; DBLASS: disharmony between liver and spleen syndrome; LQSASDS: Liver qi stagnation and spleen deficiency syndrome; LSAASS: Liver-qi stagnation and attacking spleen syndrome; WOSASS: weakness of spleen and stomach syndrome;

A: clinical therapeutic efficacy; B: TCM symptom therapeutic effect; C: abdominal pain score; D: abdominal distention score; E: abdominal discomfort score; F: diarrhea score; G: frequency of defecation score; H: mucous stool score; I: borborygmus score; J: property of stool score; K: tenesmus; L: diet condition score; M: sleeping quality score; N: physical strength; O: recurrence rate; P: IBS-QOL score; Q: IBS-BSS score; R: SCL 90; S: SF-36 scale; T: the level of serum inflammatory factor; U: the score of Hamilton Depression Rating Scale

BT: belladonna tablets; BXXX: Banxia Xiexin; CBLEABCT: combined bifidobacterium lactobacillus enterococcus and bacillus cereus tablets (Live); CGECC: compound glutamine enteric-coated capsules; DT: diazepam tablets; HXZQ: Huoxiang Zhengqi; LBP: live bifidobacterium preparation; LHS: loperamide hydrochloride capsules; MP: montmorillonite powder; OBT: otilonium bromide tablets; OT: oryzanol tablets; PBT: pinaverium bromide tablets; SB: saccharomyces boulardii sachets; SLBZS: Shenling Baizhu San; TMT: trimebutine maleate tablets; TXYF: Tongxie Yaofang; XYS: Xiayao San.

**Table 2:** Results of metaregression analysis.

Variable	Coefficient	Standard Error	t	P	95% CI
Prescription type	-0.007347	0.0163976	-0.45	0.661	0.0422977, 0.0276037
Acupuncture type	0.0071692	0.0404282	0.18	0.862	-0.0790014, 0.0933399
Treatment course	-0.0648648	0.0767705	-0.84	0.411	-0.2284971, 0.0987676
Publication year	-0.0909341	0.072595	-1.25	0.230	-0.2456667, 0.0637986

## Supplementary Materials

Supplementary 1 S1 File: a sample retrieval strategy.

Supplementary 2 S1 Table: details of acupuncture in the included trials.

Supplementary 3 S2 Table: details of CHM in the included trials.

Supplementary 4 S2 File: tests for Publication Bias.

Supplementary 5 S3 File: PRISMA checklist.
