# Supplementary material for: Acupuncture plus Chinese Herbal Medicine for Irritable Bowel Syndrome with Diarrhea: A Systematic Review and Meta-Analysis
Source: Evid Based Complement Alternat Med. 2019 Apr 14;2019:7680963. doi: 10.1155/2019/7680963 (PMC6487118; doi:10.1155/2019/7680963)
Supplement: Supplementary 1 — S1 File: a sample retrieval strategy. [file 7680963.f1.doc]

| Embase | |  |
| --- | --- | --- |
| No. | Query | Results |
| #18 | #3 AND #11 AND #14 AND #17 | **8** |
| #17 | #15 OR #16 | **509161** |
| #16 | 'controlled trial, randomized':ti,ab,kw OR 'randomised controlled study':ti,ab,kw OR 'randomised controlled trial':ti,ab,kw OR 'randomized controlled study':ti,ab,kw OR 'trial, randomized controlled':ti,ab,kw | **38026** |
| #15 | 'randomized controlled trial'/exp | **499745** |
| #14 | #12 OR #13 | **44615** |
| #13 | 'medicine, chinese traditional':ti,ab,kw OR 'traditional chinese medicine':ti,ab,kw | **20764** |
| #12 | 'chinese medicine'/exp OR 'chinese herb'/exp OR 'chinese herbal medicine'/exp | **36186** |
| #11 | #6 OR #9 OR #10 | **41928** |
| #10 | 'warm acupuncture':ti,ab,kw OR 'needle warming moxibustion':ti,ab,kw | **20** |
| #9 | #7 OR #8 | **5730** |
| #8 | 'acupuncture, electric':ti,ab,kw OR 'electric acupuncture':ti,ab,kw | **154** |
| #7 | 'electroacupuncture'/exp | **5653** |
| #6 | #4 OR #5 | **41921** |
| #5 | 'acupuncture therapy':ti,ab,kw OR auriculotherapy:ti,ab,kw OR 'point, acupuncture':ti,ab,kw | **1695** |
| #4 | 'acupuncture'/exp | **41864** |
| #3 | #1 OR #2 | **24962** |
| #2 | 'colon spasm':ti,ab,kw OR 'colon, irritable':ti,ab,kw OR 'colonic diseases, functional':ti,ab,kw OR colonospasm:ti,ab,kw OR 'irritable bowel syndrome':ti,ab,kw OR 'irritable colon syndrome':ti,ab,kw OR 'mucomembraneous colitis':ti,ab,kw OR 'mucomembranous colitis':ti,ab,kw OR'mucous colitis':ti,ab,kw OR 'spastic colitis':ti,ab,kw OR 'spastic colon':ti,ab,kw OR 'unstable colon':ti,ab,kw | **18491** |
| #1 | 'irritable colon'/exp | **22667** |

A Sample Search Strategy of Embase

Annotation:

ti: title, ab: abstract, kw: key words
