# Supplementary material for: Acupuncture plus Chinese Herbal Medicine for Irritable Bowel Syndrome with Diarrhea: A Systematic Review and Meta-Analysis
Source: Evid Based Complement Alternat Med. 2019 Apr 14;2019:7680963. doi: 10.1155/2019/7680963 (PMC6487118; doi:10.1155/2019/7680963)
Supplement: Supplementary 2 — S1 Table: details of acupuncture in the included trials. [file 7680963.f2.docx]

| **S 1 Table. The details of acupuncture in the included trials.** | | | | | | | | |
| --- | --- | --- | --- | --- | --- | --- | --- | --- |
| **Source** | **Conventional acupuncture and acupoints（international code）** | **Needle retention**  **time** | **Depth of**  **insertion** | **Needle warming moxibustion** | **Moxibus-**  **tion** | **Electro-**  **acupuncture** | **Frequency** | **Treatment sessions** |
| Shi ZM  2005 | Cheng shan (BL57) | 30 min | N.M | Chengshan (BL57) | N.M | N.M | qd | 1 month |
| Hu FL  2005 | Main acupoints: Zhongwan (RN12), Tianshu (ST25), Zusanli (ST36); Matching acupoints: Hegu (LI4), Pishu (BL20), Guanyuanshu (BL26), Shenshu (BL23), Mingmen (DU4), Ganshu (BL18), Xingjian (LR2). | N.M | N.M | MBND | N.M | N.M | qod | 2 months |
| Zhang SY 2006 | Main acupoints: Zhongwan (RN12), Tianshu (ST25), Qihai (RN6), Zhangmen (LR13),  Zusanli(ST36), Sanyinjiao(SP6), Neiguan(PC6), Taichong(SP6); Matching acupoints:  Guanyuan (RN4), Shenque (RN8), Gongsun (SP4), Neiting (ST44), Hegu(LI4),  Yinlingquan (SP9), Yanglingquan (GB34), Xuehai (SP10). | 30 min | N.M | Zhongwan (RN12),  Tianshu (ST25),  Qihai (RN6) | Guanyuan  ( RN4),  Shenque  (RN8). | N.M | qd | N.M |
| Yu YG  2007 | Zusanli (ST36), Tianshu (ST25), Shanjuxu (ST37), Xiajuxu (ST39), Sanyinjiao (SP6),  Taichong (LR3), Zhongwan (RN12), Xiawan (RN10) | 30 min | N.M | N.M | N.M | N.M | qd | 4 weeks |
| Lan YP  2010 | Main acupoints: Zhongwan (RN12), Tianshu (ST25), Jianli (RN11), Zusanli (ST36),  Shangjuxu (ST37); Matching acupoints: Pishu (BL20), Zhangmen (LR13), Ganshu (BL18), Taichong (SP6), Shenshu (BL23), Mingmen (DU4), Taixi (KI3). | 30 min | N.M | N.M | N.M | MBND | N.M | 4 weeks |
| Cao S  2011 | Zhongwan (RN12), Tianshu (ST25), Qihai (RN6), Neiguan (PC6), Zusanli (ST36),  Shangjuxu (ST37), Sanyinjiao (SP6), Gongsun (SP4), Taichong (SP6) et al. | 30 min | N.M | N.M | N.M | N.M | qd rest for 5 days,  every 15 days | 45 days |
| Tang JL 2011 | Shenjingqu(no international code), Toubuqu(no international code) in oral cavity | 15 min | 3.33 cm | N.M | N.M | N.M | qd | 4 weeks |
| Jiang QY 2013 | Zusanli (ST36), Tianshu (ST25), Shangjuxu (ST37), Sanyinjiao (SP6), Taichong (SP6),  Baihui (DU20), Yintang (EX-HN3) | 30 min | 15-30  mm | N.M | N.M | N.M | qd  6 days/week | 6 weeks |
| Jin J 2013 | Zusanli (ST36), Tianshu (ST25), Shangjuxu (ST37), Sanyinjiao (SP6), Taichong (SP6),  Baihui (DU20), Yintang (EX-HN3) | N.M | N.M | N.M | N.M | N.M | qd  5 days/week | 4 weeks |
| Zhou P 2014 | Zusanli (ST36), Tianshu (ST25) | MBND | 3.33-4.95 cm | Zusanli (ST36),  Tianshu (ST25) | N.M | N.M | qod | 8 weeks |
| Yan YZ 2014 | Tianshu (ST25), Shangjuxu (ST37), Sanyinjiao (SP6), Taichong (LR3), Zusanli (ST36) | 30 min | 20-25 mm | N.M | N.M | N.M | qd  5 days/week | 4 weeks |
| Xu SC 2015 | Tianshu (ST25), Zusanli (ST36), Shanjuxu (ST37), Taichong (LR3), Zhongwan (RN12),  Ganshu (BL18),Pishu (BL20), Yinlingquan (SP9),Shenshu (BL23), Mingmen (DU4),  Xingjian(LR2), Shenguan (no international code) | 45min | N.M | N.M | N.M | N.M | qd | 10 days |
| Li YX 2015 | Zusanli (ST36), Tianshu (ST25) | 30 min | 25-37 mm | Zusanli (ST36), Tianshu (ST25) | N.M | N.M | qod | 8 weeks |
| Sun W 2015 | Shenjingqu (no international code), Toubuqu (no international code) in oral cavity | 15 min | 3.3 cm | N.M | N.M | N.M | qd | 4 weeks |
| Zhi YC 2016 | Tianshu (ST25), Zusanli (ST36), Shangjuxu (ST37), Sanyinjiao (SP6), Taichong (LR3),  Baihui (DU20), Yintang (EX-HN3) | 30 min | N.M | N.M | N.M | N.M | qd  5 days/week | 4 weeks |
| Wang XH 2016 | Tianshu (ST25), Zusanli (ST36), Yinlingquan (SP9), Xiajuxu (ST39), Taichong (LR3) | 30 min | N.M | N.M | N.M | N.M | qd | 1 month |
| Chen S 2016 | Tianshu (ST25), Zusanli (ST36), Shangjuxu (ST37), Taichong (SP6) | 20 min | N.M | N.M | N.M | Tianshu (ST25),  Zusanli (ST36), Shangjuxu (ST37),  Tai chong (SP6) | qd | 4 weeks |
| Yang JY 2017 | Tianshu (ST25), Zusanli (ST36), Shangjuxu (ST37), Sanyinjiao (SP6), Taichong (LR3),  Baihui (DU20) | 30 min | N.M | N.M | N.M | Tianshu (ST25),  Zusanli (ST36), Shangjuxu (ST37) | qd  5 days/week | 4 weeks |
| Wang W 2017 | Wei acupoint (no international code), Xin acupoint (no international code), Gan acupoint (no international code), Pi acupoint (no international code) in forearm skin | 30 min | N.M | N.M | N.M | N.M | qd | 1 month |
| Hou GH 2017 | Pishu (BL20), Tianshu (ST25), Yinlingquan (SP9), Sanyinjiao (SP6), Taichong (LR3),  Shangjuxu (ST37), Zusanli (ST36) et al. | 30 min | 20-25 mm | N.M | N.M | N.M | qd | 4 weeks |
| Sun M 2017 | Tianshu (ST25), Zusanli (ST36), Xiajuxu (ST39), Taichong (LR3), Yinlingquan (SP9) | 30 min | N.M | N.M | N.M | N.M | qd | 2 weeks |

Annotation:

N.M: not mentioned; qd: once a day; qod: every other day
