# Supplementary material for: Acupuncture plus Chinese Herbal Medicine for Irritable Bowel Syndrome with Diarrhea: A Systematic Review and Meta-Analysis
Source: Evid Based Complement Alternat Med. 2019 Apr 14;2019:7680963. doi: 10.1155/2019/7680963 (PMC6487118; doi:10.1155/2019/7680963)
Supplement: Supplementary 3 — S2 Table: details of CHM in the included trials. [file 7680963.f3.docx]

| **S2_Table. Details of CHM in the included trials.** | | | | |
| --- | --- | --- | --- | --- |
| **Source** | **Content**(***Chinese PinYin name, Latin Herb name***) | **Preparationstyle** | **Dosage and administration** | **Treatment session** |
| Shi ZM 2005 | Basic recipe: Zhishi (A*urantii Fructus Immaturus*) 10 g, Baishao (*Paeoniae Radix Alba*) 20 g, Chaihu (*Bupleuri Radix*) 12 g, Chenpi (*Citri Reticulatae Pericarpium*) 10 g, Baizhu (*Atractylodis Macrocephalae Rhizoma*) 20 g, Zhigancao (*Glycyrrhizae Radix Et Rhizoma Praeparatata Cum Melle*) 6g, Fangfeng (*Saposhnikoviae Radix*) 10 g. Modified: (1) Yanhusuo (*Corydalis Rhizoma* ) 12g, Muxiang (*Aucklandiae Radix*) 12g; (2) Wumei (*Mume Fructus*) 15 g, Roudoukou (*Myristicae Semen*) 15 g. | decoction | 1 dose,  bid, po | 1 month |
| Hu FL 2005 | Basic recipe: Baishao (*Paeonia lactifora*) 20 g, Baizhu (*Atractylodis Macrocephalae Rhizoma*) 12 g, Yujin (*Curcumae Radix*) 12 g, Zhigancao (*Glycyrrhizae Radix Et Rhizoma Praeparatata Cum Melle*) 12 g, Fangfeng (*Saposhnikoviae Radix*) 10 g, Chenpi (*Citri Reticulatae Pericarpium*) 6 g. Modified: (1)Taizishen (*Pseudostellariae Radix*) 15 g, Gegen (*Puerariae Lobatae Radix*) 12 g; (2) Chaihu ((*Bupleuri Radix*) 15 g, Zhiqiao (*Aurantii Fructus*) 10 g; (*3*) Buguzhi (*Psoraleae Fructus*) 10 g, Wuzhuyu (*Euodiae Fructus*) 5 g, Wuweizi (*Schisandrae chinensis Fructus*) 6 g; (*4*) Zhenzhumu (*Margaritifera Concha*) 30 g, Baihe (*Lilii Bulbus*) 30 g, Xiaomai (*Hordei Fructus Germinatus*) 30 g. | decoction | 1 dose,  tid, po | 2 months |
| Zhang SY 2006 | Oral basic recipe: Chaihu (*Bupleuri Radix*) 15 g, Baishao (*Paeoniae Radix Alba*) 12 g, Sutiaoshen(*Glehniae Radix*) 15 g, Fuling (*Poria*) 15 g, Baizhu (*Atractylodis Macrocephalae Rhizoma*) 12 g, Cangzhu (*Atractylodis Rhizoma* )15 g, Chenpi (*Citri Reticulatae Pericarpium*) 8 g, Yuanhu (*Corydalis Rhizoma* ) 10 g, Yiyiren(*Coicis Semen*) 30 g, Cheqianzi (*Plantaginis Semen*) 10 g, Guangmuxiang (*Aucklandiae Radix*) 6 g, Sharen (*Amomi Fructu*) 10 g, Houpo (*Magnoliae officinalis Cortex*) 8 g, Gancao (*Glycyrrhizae Radix Et Rhizoma Praeparatata Cum Melle*) 3 g. Oral modified: (*1*) Paojiang (*Zingiberis Rhizoma Praeparatum*) 6 g, Wuyu (*Psoraleae Fructus*) 6 g, Xiaohuixiang (*Foeniculi Fructus*) 10 g; (*2*) Chaoguya (*Setariae Fructus Germinatus*) 15 g, Chaomaiya (*Hordei Fructus Germinatus*)15 g, Jineijin (*Galli Gigerii Endothelium Corneum*) 10 g; (*3*) Huanglian (*Coptidis Rhizoma*) 6 g, Binlang (*Arecae Semen*)10 g; (*4*) Chishao (*Paeoniae Radix Rubra*) 10 g, Danggui (*Angelicae sinensis Radix*) 15 g, Chuanlianzi (*Toosendan Fructus*) 10 g, Shengshanzha (*Crataegui Fructus*) 10 g. Coloclysis recipe: Huanglian (*Coptidis Rhizoma.*) 20 g, Baiji (*Bletillae Rhizoma*) 20 g, Huangqi (*Astragali Radix*) 30 g, Diyu (*Sanguisorbae Radix*) 15 g, Chishao (*Paeoniae Radix Rubra*) 15 g, Baishao (*Paeonia lactifora*) 15 g. | decoction | Oral administration:  1 dose, tid  enema therapy: 50ml, qd | N.M |
| Yu YG 2007 | Huanglian ( *Coptidis Rhizoma*), Cangzhu (*Atractylodis Rhizoma*), Baiji (*Bletillae Rhizoma*), Yingsuqiao (*Papaveris Pericarpium*). | capsule | 6 capsules,  tid, po | 4 weeks |
| Lan YP 2010 | Basic recipe: Baizhu (*Atractylodis Macrocephalae Rhizoma*) 20 g, Baishao (*Paeonia lactifora*) 15 g, Chenpi (*Citri Reticulatae Pericarpium*)10 g, Zhiqiao (*Aurantii Fructus*) 10 g, Fangfeng (*Saposhnikoviae Radix*) 10 g, Shengma (*Cimicifugae Rhizoma*) 5 g, Chaihu (*Bupleui Radix*) 5 g, Weihezi (*Chebulae Fructus*) 10 g, Xiangfu (*Cyperui Rhizoma*) 10 g, Chuanxiong (*Chuanexiong Rhizoma*) 9 g. Modified: (1) Fuling (*Poria*) 15 g, Yiyiren (*Coicis Semen*) 30 g; (2) Wuzhuyu (*Psoraleae Fructus*) 10 g, Paojiang (*Zingiberis Rhizoma Praeparatum*) 10 g, Buguzhi (*Psoraleae Fructus*) 10 g, Wuzhuyu (*Psoraleae Fructus*) 12 g; (3) Jineijin (*Galli Gigerii Endothelium Corneum*) 10 g, Jiaoshanzha (*Crataegui Fructus*) 15 g, Shenqu (*Massa Medicata Fermentata*)15 g; (4) Dangshen (*Codonopsis Radix*) 20 g, Shanyao (*Dioscoreae Rhizoma*) 15 g; (*5*) Huanglian (*Coptidis Rhizoma*) 5 g, Huangqin (*Scutellariae Radix*) 15 g, Machixian (*Portulacae Herba*) 20g. | decoction | 1 dose, bid, po | 4 weeks |
| Cao S 2011 | Huoxiang (*Pogostemonis Herba*), Baizhu (*Atractylodis Macrocephalae Rhizoma*), Houpu (*Magnoliae officinalis Cortex*), Fabanxia (Pinelliae Rhizoma *Praeparatum*), Zisuye (*Perillae Folium*), Baizhi (*Angelicae dahuricae Radix*), Chenpi (*Citri Reticulatae Pericarpium*), Fuling (*Poria*), Jiegeng (*Platycodonis Radix)*, Gancao (*Glycyrrhizae Radix Et Rhizoma*), Shengjiang (*Zingiberis Rhizoma Recens*), Dazao (*Jujubae Fructus*). | pill or [liquid](C:/Users/ibm/AppData/Local/Youdao/Dict/Application/7.5.0.0/resultui/dict/?keyword=liquid) or soft capsule,  or drop pill | (1) pill: 8 pills,  tid, po; (2) [oral](C:/Users/ibm/AppData/Local/Youdao/Dict/Application/7.5.0.0/resultui/dict/?keyword=oral)[liquid](C:/Users/ibm/AppData/Local/Youdao/Dict/Application/7.5.0.0/resultui/dict/?keyword=liquid):10ml, tid, po; (3) [soft](C:/Users/ibm/AppData/Local/Youdao/Dict/Application/7.5.0.0/resultui/dict/?keyword=soft)[capsule](C:/Users/ibm/AppData/Local/Youdao/Dict/Application/7.5.0.0/resultui/dict/?keyword=capsule): 2-4 capsules, tid, po; (4) drop pill:  1-2 bags, bid, po | 45 days |
| Tang JL 2011 | Basic recipe: Shenghuangqi (*Astragali Radix*) 20 g, Sharen (*Amomi Fructus*) 15 g, Jiangcan (*Bombyx Batryticatus*) 15 g, Quanxie (*Scorpio*) 6 g, Huoxiang (*Pogostemonis Herba*) 15 g, Baishao (*Paeonia lactifora*) 10 g, Baizhu (*Atractylodis Macrocephalae Rhizoma*) 10 g, Chenpi (*Citri Reticulatae Pericarpium*)10 g, Fangfeng (*Saposhnikoviae Radix*)10 g. Modified: (1) Baishao (*Cynanchum otophyllum*) 15 g, Gancao (*Glycyrrhizae Radix Et Rhizoma*) 10 g; (2) Yiyiren (*Coicis Semen*) 15 g, Cangzhu (*Atractylodis Rhizoma*) 10 g; (3) Huangqin (*Scutellariae Radix*)10 g; (4) Guizhi (*Cinnamomi Ramulus*) 10 g, Ganjiang (*Zingiberis Rhizoma*) 8 g. | decoction | 1 dose, bid, po | 4 weeks |
| Jiang QY 2013 | Basic recipe: Chaihu (*Bupleui Radix*) 12 g, Chenpi (*Citri Reticulatae Pericarpium*) 6 g, Chuanxiong (*Chuanexiong Rhizoma*) 9 g, Xiangfu (*Cyperui Rhizoma*) 6 g, Zhiqiao (*Aurantii Fructus*) 9 g, Baishao (*Paeonia lactifora*) 9 g, Zhigancao (*Glycyrrhizae Radix Et Rhizoma Praeparatata Cum Melle*) 6 g, Baizhu (*Atractylodis Macrocephalae Rhizoma*) 12g, Fuling (*Poria*) 12 g, Fangfeng (*Saposhnikoviae Radix*) 9 g. Modified: (1) Chenxiang (*Aquilariae Lignum Resinatum*), Baikouren (*Amomi Fructus Rotundus*); (2) Dangshen (*Codonopsis Radix*), Wumei (*Mume Fructus*), Mugua (*Chaenomelis Fructus*); (3) Zaoren (*Ziziphi Spinosae Semen*), Yejiaoteng (*Polygoni Multiflori Caulis*) | decoction | 1 dose, bid, po | 6 weeks |
| Jin J 2013 | Basic recipe: Baishao (*Paeonia lactifora*) 10 g, Baizhu (*Atractylodis Macrocephalae Rhizoma*)10 g, Chenpi (*Citri Reticulatae Pericarpium*) 10 g, Fangfeng (*Saposhnikoviae Radix*) 10 g, Dangshen (*Codonopsis Radix*) 15 g, Fuling (*Poria*) 15 g, Xiangfu (*Cyperui Rhizoma*) 10 g, Chaihu (*Bupleui Radix*) 10 g, Muixang (*Aucklandiae Radix*) 10 g, Zhiqiao (*Aurantii Fructus*) 10g , Zhigancao (*Glycyrrhizae Radix Et Rhizoma Praeparatata Cum Melle*) 6 g. Modified: (1) Huanglian (*Coptidis Rhizoma.*); (2) Yuanhu (Corydalis Rhiz*oma*). | decoction | 1 dose, qd, po | 1 month |
| Zhou P 2014 | Chaihu (*Bupleurum chinense*) 15 g, Danggui (*Angelicae sinensis Radix*) 10 g, Baishao (*Paeonia lactifora*) 10g, Baizhu (*Atractylodis Macrocephalae Rhizoma*) 10g, Fuling (*Poria*) 10 g, Gaocao (*Glycyrrhizae Radix Et Rhizoma*) 5 g, Dangshen (*Codonopsis Radix*) 10 g, Bohe (*Menthae Haplocalycis Herba*) 5 g, Xiangfu (*Cyperui Rhizoma*) 10 g, Yujin (*Curcumae Radix*) 10 g, Chenpi (*Citri Reticulatae Pericarpium*) 10 g, Zhiqiao (*Aurantii Fructus*) 10 g, Wumei (*Mume Fructus*) 10 g, Wuweizi (*Schisandrae chinensis Fructus*) 10 g*,* Shiliupi (*Granati Pericarpium*) 10 g, Shanzha (*Crataegui Fructu*) 10 g, Maiya (*Hordei Fructus Germinatus*) 10 g, Shenqu (*Massa Medicata Fermentata*) 10 g | decoction | 1 dose, bid, po | 8 weeks |
| Yan YZ 2014 | Basic recipe: Chaihu (*Bupleui Radix*) 12 g, Baishao (*Paeonia lactifora*) 20 g, Foshou (*Citri Sarcodactylis Fructus*) 10 g, Xiangyuanpi (*Citri Fructus*) 10 g, Huangqi (*Astragali Radix*) 12 g, Taizishen (*Pseudostellariae Radix* ) 12 g, Baizhu (*Atractylodis Macrocephalae Rhizoma*) 15 g, Fuling (*Poria*) 10 g, Gegen (*Puerariae Lobatae Radix*) 12 g, Chenpi (*Citri Reticulatae Pericarpium*) 10 g, Fangfeng (*Saposhnikoviae Radix*) 6 g, Zhigancao (*Glycyrrhizae Radix Et Rhizoma Praeparatata Cum Melle*) 10 g. Modified: (1) Qingpi (*Citri reticulatae Pericarpium Viride*) 10 g, Xiangfu (*Cyperui Rhizoma*) 10 g; (2) Yujin (*Curcumae Radix*) 12 g, Shanzhizi (*Gardeniae Fructus*) 12 g; (3) Zhiqiao (*Aurantii Fructus*) 12 g, Houpu (*Magnoliae officinalis Cortex*)10 g; (4) biandou (*Lablab Semen Album*)15 g, Cangzhu (*Atractylodis Rhizoma*) 10 g, Faxia (*Pinelliae Rhizoma Praeparatum*) 10 g; (5) Danggui *Angelicae sinensis Radix*) 15 g; (6) Lujiaoshuang (*Cervi Cornu Degelatinatum*) 12 g, Roudoukou (*Myristicae Semen.*) 6 g, Buguzhi (*Psoraleae Fructus*) 12 g | decoction | 1 dose, bid, po | 4 weeks |
| Xu SC 2015 | Basic recipe: Baishao (*Paeonia lactifora*) 15 g, Baizhu (*Atractylodis Macrocephalae Rhizoma*) 10 g, Chenpi (*Citri Reticulatae Pericarpium*) 10 g, Fangfeng (*Saposhnikoviae Radix*) 10 g, Yiyiren ( *Coicis Semen*) 10 g, Zhiqiao ( *Aurantii Fructus*) 10 g, Fuling (*Poria*) 12 g, Gancao (*Glycyrrhizae Radix Et Rhizoma*) 5 g. Modified: (1)Biandou (*Lablab Semen Album*) 10 g, Wumei (*Mume Fructus*) 10 g; (2) Maiya (*Hordei Fructus Germinatus*) 10 g, Shanzha (*Crataegui Fructus*) 10 g, Shenqu (*MassaMedicata Fermentata*) 10 g, Jineijin (*Galli Gigerii Endothelium Corneum*) 15 g; (3) Houpo (*Magnoliae officinalis Cortex*) 10 g, Baikou (*Amomi Fructus Rotundus*) 10g, Muxiang (*Aucklandiae Radix*) 9 g; (4) Chuanlianzi (*Toosendan Fructus*) 10 g, Yujin (*Curcumae Radix*) 8 g; (5) Dangshen (*Codonopsis Radix*) 10 g, Shenghuangqi (*Astragali Radix*) 15 g. | decoction | 1 dose, bid, po | 10 days |
| Li YX 2015 | Chaihu (*Bupleurum chinense*) 15 g, Danggui (*Angelicae sinensis Radix*) 10 g, Baishao (*Paeonia lactifora*) 10 g, Baizhu (*Atractylodis Macrocephalae Rhizoma*) 10 g, Fuling (*Poria*) 10 g, Dangshen (*Codonopsis Radix*) 10 g, Bohe (*Menthae Haplocalycis Herba*) 5 g, Xiangfu (*Cyperui Rhizoma*) 10 g, Yujin (*Curcumae Radix*) 10 g, Chenpi (*Citri Reticulatae Pericarpium*) 10 g, Zhiqiao (*Aurantii Fructus*) 10 g, Wumei (*Mume Fructus*) 10 g, Wuweizi (*Schisandrae chinensis Fructu*s) 10 g, Shiliupi (*Granati Pericarpium*) 10 g, Shanzha (*Crataegui Fructu*) 10 g, Maiya (*Hordei Fructus Germinatus*) 10 g, Shenqu (*Massa Medicata Fermentata*) 10 g | decoction | 1 dose, bid, po | 8 weeks |
| Sun W 2015 | Shenghuangqi (*Astragali Radix*) 20 g, Sharen (*Amomi Fructus*) 15 g, Jiangcan (*Bombyx Batryticatus*) 15 g, Baizhu (*Atractylodis Macrocephalae Rhizoma*) 10 g, Baishao (*Paeonia lactifora*) 10 g, Huoxiang (*Pogostemonis Herba*) 15 g, Chenpi (*Citri Reticulatae Pericarpium*) 10 g, Fangfeng (*Saposhnikoviae Radix*) 10 g, Quanxie (*Scorpio*) 6 g. | decoction | 1 dose, bid, po | 4 weeks |
| Zhi YC 2016 | Shanyao (*Dioscoreae Rhizoma*) 30 g, Fuling (*Poria*) 30 g, Baishao (*Paeonia lactifora*) 24 g, Shudi (*Rehmanniae Radix Praeparata*) 24 g, Chaihu (*Bupleui Radix*) 15 g, Baizhu (*Atractylodis Macrocephalae Rhizoma*) 15 g, Ganjiang (*Zingiberis Rhizoma*) 9 g, Zhigancao (*Glycyrrhizae Radix Et Rhizoma Praeparatata Cum Melle*) 9 g, Chenpi (*Citri Reticulatae Pericarpium*) 9 g, Fangfeng (*Saposhnikoviae Radix*) 6 g. | decoction | 1 dose, bid, po | 4 weeks |
| Wang XH 2016 | Basic recipe: Banxia (*Pinelliae Rhizoma*) 12 g, Wumei (*Mume Fructus*) 12 g, Huangqin (*Scutellariae Radix*) 12 g, Wuzhuyu (*Psoraleae Fructus*)10 g, Ganjiang (*Zingiberis Rhizoma*) 9 g, Zhigancao ( *Glycyrrhizae Radix Et Rhizoma Praeparatata Cum Melle*) 6 g, Huanglian ( *Coptidis Rhizoma*) 6 g, Dazao (*Jujubae Fructus*) 2, Baizhu (*Atractylodis Macrocephalae Rhizoma*)15 g, Dangshen (*Codonopsis Radix*) 12 g. Modified: (1) Yanhusuo (Corydalis Rhiz*oma*) 12 g; (2) Muxiang (*Aucklandiae Radix*) 12 g, Zhishi (*Auranti Fructus Immaturus*)10 g; (3) Huoxiang (*Pogostemonis Herba*) 10 g, Cangzhu (*Atractylodis Rhizoma* ) 12 g; (4) Roudoukou (*Myristicae Semen*) 12 g, Buguzhi (*Psoraleae Fructus*) 12 g; (5) Chaihu (*Bupleui Radix*) 12 g, Xiangfu (*Cyperui Rhizoma*) 12 g; (6) Shudi (*Rehmanniae Radix Praeparata*) 15 g, Huangqi (*Astragali Radix*) 15 g. | decoction | 1 dose, bid, po | 2 weeks |
| Chen S 2016 | Basic recipe: Chaihu (*Bupleui Radix*) 10 g, Dangshen (*Codonopsis Radix*) 15 g, Fuling (*Poria*) 15 g, Baizhu (*Atractylodis Macrocephalae Rhizoma*) 10 g, Baishao (*Paeonia lactifora*) 10 g, Foshou (*Citri sarcodactylis Fructus*) 10 g, Baibiandou( *Lablab Semen Album*) 10 g, Buguzhi (*Psoraleae Fructus*) 10 g Wuzhuyu (*Psoraleae Fructus*) 10 g. Modified: (1) Huangqin ( *Scutellariae Radix*), Yiyiren (*Coicis Semen*); (2) Rougui (*Cinnamomi Cortex*); (3)Yanhusuo (Corydalis Rhiz*oma*), Chuanlianzi (*Toosendan Fructus*). | decoction | 1 dose, bid, po | 4 weeks |
| Yang JY 2017 | Dangshen (*Codonopsis Radix*) 15 g, Baizhu (*Atractylodis Macrocephalae Rhizoma*) 15 g, Fuling (*Poria*) 15 g, Shanyao (*Dioscoreae Rhizoma*) 15 g, Yiyiren (*Coicis Semen*) 30 g, Baibiandou (*Lablab Semen Album*) 10 g, Sharen (*Amomi Fructus*) 10 g, Fangfeng *Saposhnikoviae Radix*) 10 g, Jiegeng (*Platycodonis Radix*) 10 g, Zhigancao (*Glycyrrhizae Radix Et Rhizoma Praeparatata Cum Melle*) 6 g, Lianrou (*Nelumbinis Semen*) 10 g, Huoxiang (*Pogostemonis Herba*)10 g, Chenpi (*Citri Reticulatae Pericarpium*) 10 g, Qingpi (*Citri reticulatae Pericarpium Viride*) 10 g, Ganjiang (*Zingiberis Rhizoma*) 5 g, Baishao (*Paeonia lactifora*) 10 g, Rougui (*Cinnamomi Cortex*) 6 g. | N.M | N.M | 4 weeks |
| Wang W 2017 | Chenpi (*Citri Reticulatae Pericarpium*) 12 g, Baishao (*Paeonia lactifora*) 20 g, Baizhu (*Atractylodis Macrocephalae Rhizoma.*) 20 g, Yiyiren ( *Coicis Semen*) 30 g, Beichaihu (*Bupleui Radix*) 6 g, Guanghuoxiang (*Pogostemonis Herba*) 12 g, Dangshen (*Codonopsis Radix*) 10 g, Baibiandou (*Lablab Semen Album*) 12 g, Sharen (*Amomi Fructus*) 8 g, Hehuanpi (*Albiziae Cortex*) 20 g, Fuling (*Poria*) 20 g, Shouwuteng (*Polygoni Multiflori Caulis* ) 20 g. | decoction | 4 capsules,  tid, po | 1 month |
| Hou GH 2017 | Baizhu (*Atractylodis Macrocephalae Rhizoma*), Fuling (*Poria* ), Gaocao (*Glycyrrhizae Radix Et Rhizoma.*), L-Glutamine | capsule | 1dose, bid, po | 4 weeks |
| Sun M 2017 | Basic recipe: Wumei (*Mume Fructus*) 12 g, Banxia (*Pinelliae Rhizoma*) 12 g, Huangqin (*Scutellariae Radix*) 12 g, Gancao (*Glycyrrhizae Radix Et Rhizoma*) 6 g, Huanglian (*Coptidis Rhizoma*) 6 g, Wuzhuyu (*Psoraleae Fructus*) 10 g, Dazao (*Jujubae Fructus*) 2, Ganjiang (*Zingiberis Rhizoma*) 9 g, Dangshen (*Codonopsis Radix*) 12 g. Modified: (1) Yanhusuo (Corydalis Rhiz*oma*) 12 g; (2) Muxiang (*Aucklandiae Radix*) 12 g, Zhishi (*Auranti Fructus Immaturus*)10 g; (3)Buguzhi (*Psoraleae Fructus*) 12 g, Roudoukou (*Myristicae Semen*)12 g; (4) Huoxiang (*Pogostemonis Herba*)10 g, Cangzhu (*Atractylodis Rhizoma* )12 g; (5) Chaihu (*Bupleui Radix*) 12 g, Xiangfu (*Cyperui Rhizoma*)12 g; (6) Huangqi (*Astragali Radix*) 15 g, (*Rehmanniae Radix Praeparata*) 15 g | decoction | 1 dose, bid, po | 2 weeks |

Annotation:

qd: once a day; bid: twice a day; tid: three times a day; po: [oral](C:/Users/ibm/AppData/Local/Youdao/Dict/Application/7.5.0.0/resultui/dict/?keyword=oral)[administration](C:/Users/ibm/AppData/Local/Youdao/Dict/Application/7.5.0.0/resultui/dict/?keyword=administration); CGECC: compound glutamine enteric-coated capsules; N.M: not mentioned
