# Supplementary material for: Acupuncture plus Chinese Herbal Medicine for Irritable Bowel Syndrome with Diarrhea: A Systematic Review and Meta-Analysis
Source: Evid Based Complement Alternat Med. 2019 Apr 14;2019:7680963. doi: 10.1155/2019/7680963 (PMC6487118; doi:10.1155/2019/7680963)

Tests for Publication Bias

Egger's test

------------------------------------------------------------------------------

Std_Eff | Coef. Std. Err. t P>|t| [95% Conf. Interval]

-------------+----------------------------------------------------------------

slope | -.0531948 .04091 -1.30 0.210 -.1391435 .032754

bias | 3.076067 .44405 6.93 0.000 2.143153 4.008981

------------------------------------------------------------------------------


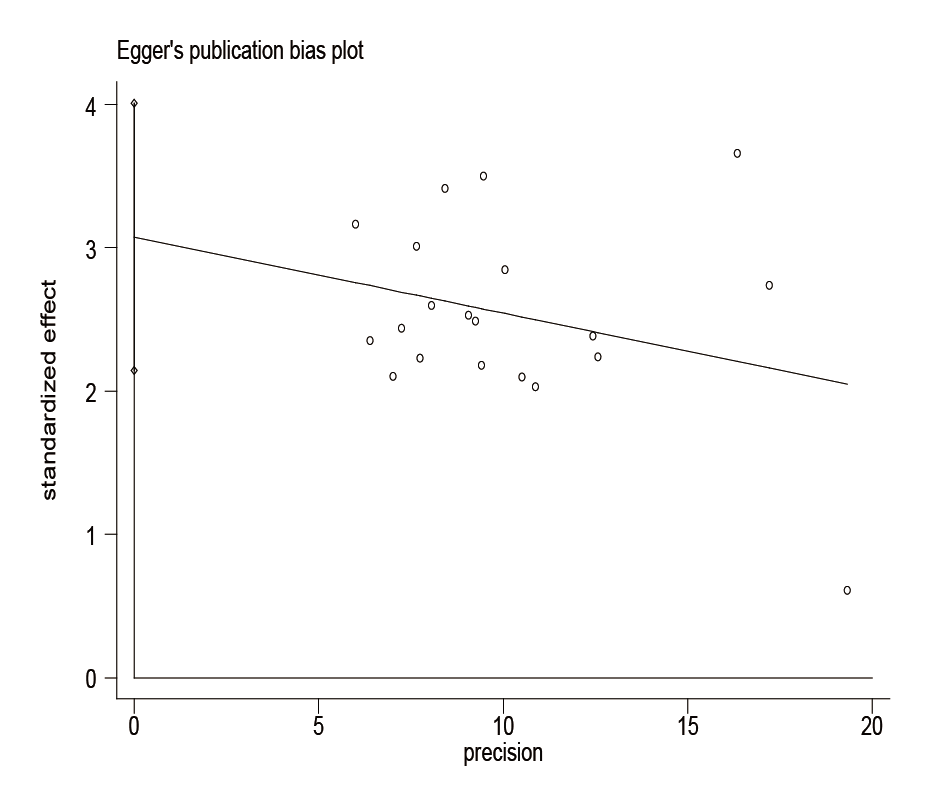


. metatrim _ES _selogES, graph funnel

Note: default data input format (theta, se_theta) assumed.

Meta-analysis

| Pooled 95% CI Asymptotic No. of

Method | Est Lower Upper z_value p_value studies

-------+----------------------------------------------------

Fixed | 1.247 1.206 1.287 60.511 0.000 20

Random | 1.295 1.229 1.361 38.423 0.000

Test for heterogeneity: Q= 44.826 on 19 degrees of freedom (p= 0.001)

Moment-based estimate of between studies variance = 0.012

Trimming estimator: Linear

Meta-analysis type: Fixed-effects model

iteration | estimate Tn # to trim diff

----------+--------------------------------------

1 | 1.247 167 6 210

2 | 1.206 188 9 42

3 | 1.191 194 9 12

4 | 1.191 194 9 0

Filled

Meta-analysis

| Pooled 95% CI Asymptotic No. of

Method | Est Lower Upper z_value p_value studies

-------+----------------------------------------------------

Fixed | 1.191 1.155 1.228 63.923 0.000 29

Random | 1.205 1.136 1.273 34.502 0.000

Test for heterogeneity: Q= 88.351 on 28 degrees of freedom (p= 0.000)

Moment-based estimate of between studies variance = 0.022


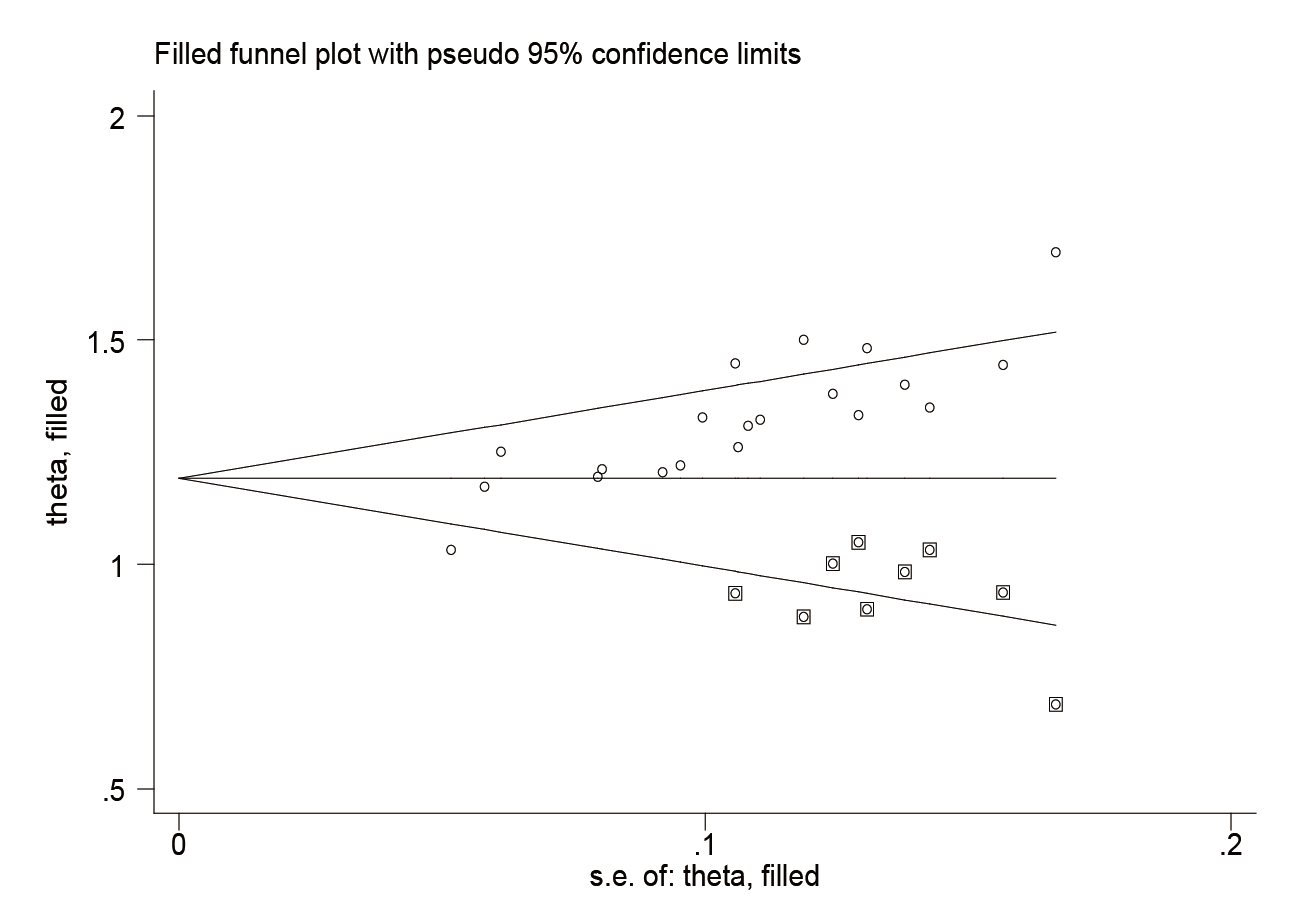

Supplement: Supplementary 4 — S2 File: tests for Publication Bias. [file 7680963.f4.docx]
